# Impact of the age at menarche on body composition in adulthood: results from two birth cohort studies

**DOI:** 10.1186/s12889-016-3649-x

**Published:** 2016-09-22

**Authors:** Susana Bubach, Ana Maria Baptista Menezes, Fernando Celso Barros, Fernando César Wehrmeister, Helen Gonçalves, Maria Cecília Formoso Assunção, Bernardo Lessa Horta

**Affiliations:** 1 Departament of Health Sciences, Federal University of Espirito Santo, São Mateus, Espirito Santo Brazil; 2 Postgraduate Programme in Epidemiology, Federal University of Pelotas, Rua Marechal Deodoro, 1160, 3° piso, Centro, Pelotas, Rio Grande do Sul Cep: 96020-220 - Caixa Postal 464 Brazil; 3 Postgraduate Programme in Health and Behavior, Catholic University of Pelotas, Pelotas, Rio Grande do Sul Brazil

**Keywords:** Menarche, Body composition, Adult, Adolescence, Epidemiology

## Abstract

**Background:**

Evidence suggests that early menarche is positively associated with adiposity in adulthood. However, it is important to assess whether this association is due to early menarche or to the association of adiposity in late childhood with age at menarche. We evaluated the association between age at menarche and body composition in adolescence and adulthood, among subjects who have been prospectively followed in two Brazilian birth cohort studies.

**Methods:**

In 1982 and 1993, the hospitals births in Pelotas were identified, and these subjects have been followed for several times. Information on age at menarche was obtained from the women (1982 cohort) and their mothers (1993 cohort). At 30 and 18 years, the following body composition measures were evaluated: body mass index, waist circumference, fat-free mass index and fat mass index measured by dual-energy x-ray absorptiometry, and thickness of the abdominal visceral fat layer measured by ultrasound. The analyses were adjusted for: birth weight, maternal pregestational weight, gestational age, family income, household score index, maternal schooling, weight-for-height z-score at 4 years (1982), and body mass index at 11 years (1993).

**Results:**

At 30 and 18 years, 2045 and 2092 women were evaluated, respectively. The prevalence of early menarche (≤11 years of age) was 24.7 % in the 1982 and 27.6 % in the 1993 cohort. In the 1982 cohort, early menarche was positively associated with all body composition variables compared to those with late menarche (≥14 years of age) even after adjusting for confounders (fat mass index: 2.33 kg/m^2^, 95 % Confidence interval: 1.64; 3.02). However, in the 1993 cohort, after adjusting for body mass index at 11 years, the regression coefficient for the association with fat mass index decreased from 2.2 kg/m^2^ (95 % Confidence interval: 1.7; 2.6) to 0.26 (95 % Confidence interval: −0.08; 0.60).

**Conclusions:**

The association between age at menarche and body composition in adulthood is strongly explained by pre-pubertal adiposity.

## Background

Menarche is a landmark in female sexual maturation [1], occurring in late puberty after intense physical and metabolic changes [2–4]. During female sexual maturation, fat is progressively deposited in subcutaneous tissues, particularly in the region of the hip, due to the effect of estrogen [5, 6], which increases body mass index (BMI) from 0.5 to 1.3 kg/m^2^ as the women goes through the Tanner maturation stages [7].

It has been reported a reduction in age at menarche in most countries. For example, MacDoweel et al. [8], in the National Health and Nutrition Examination Survey (NHANES) in the United States of America, observed that the mean age at menarche decreased from 13.3 years for women born prior to 1920 to 12.4 years among those who were born between 1980 and 1984. A decrease in age at menarche has also been observed in European countries [9].

Evidence suggests that early menarche is associated with greater adiposity in adulthood. A meta-analysis [10] observed that early menarche (≤11 years of age) increased the risk of obesity in adulthood [pooled odds ratio (OR): 2.00; 95 % confidence interval (CI): 1.79; 2.24].

Furthermore, it has also been reported that early menarche would be associated with a higher risk of chronic diseases such as cancer, diabetes, and cardiovascular disease [11–15].

On the other hand, this association between age at menarche and chronic diseases may be due to adiposity in childhood, since age at menarche is inversely associated with BMI in childhood [16]. Therefore, age at menarche would be a marker of weight gain in childhood. Indeed, Pierce et al. [17] observed in the 1946 British cohort that the magnitude of the association between age at menarche and BMI at 53 years decreased and the confidence interval included the null value after adjusting for BMI at 11 years (β adjusted: −0.23; 95 % CI: −0.50; 0.04).

This study was aimed at assessing the association between age at menarche and body composition (BMI, waist circumference, fat and fat-free mass indices, and abdominal visceral fat thickness) in adolescence and adulthood among women who have been prospectively followed since birth in Pelotas, a southern Brazilian city. In order to evaluate whether adiposity in late childhood confounds this association, in the 1993 cohort, the analyses will be adjusted for BMI at 11 years.

## Methods

This study is based on data from two birth cohort studies carried out in Pelotas, a southern Brazilian city. In 1982 and 1993, all maternity hospitals in the city were visited daily, and the births were identified. Those liveborns whose families lived in the urban area of the city were examined, and their mothers interviewed soon after delivery. These individuals have been followed for several times [18–20]. Between June 2012 and February 2013 (mean age of 30.2 years), an attempt was made to follow all participants of the 1982 cohort [20]. A similar attempt was made for the 1993 cohort between September 2011 and March 2012 (mean age of 18.5 years) [19].

In the 1982 cohort, information on age at menarche was obtained from the women in the 23 years follow-up visit. In the 1993 cohort, information on the age at menarche was obtained from mother in the 15 years visit. Age at menarche was assessed as the age of occurrence of the first menstrual cycle.

In the 2012–13 and 2011–12 visits of the 1982 and 1993 cohort, respectively, the cohort members were invited to visit the research clinic to be interview and examined. Weight was measured using the Bod Pod scale and height a portable stadiometer (accuracy of 0.1 cm). Waist circumference (in cm) was measured with the subject standing, with the arms hanging freely and next to the body, using a non-elastic measuring tape in the horizontal plane around the narrowest part of the waist. In obese subjects, the measure was taken in the horizontal plane at the point between the last rib and the iliac crest. Fat-free and fat mass (kg/m^2^) were assessed using dual-energy x-ray absorptiometry (DXA) and fat-free and fat mass indexes were estimated by dividing fat-free and fat mass by height square. Overweight was defined as a BMI of 25 kg/m^2^ or more. In the 1982 cohort, the thickness of the abdominal visceral fat layer (in cm) was measured using ultrasound while the subjects were laying in the supine position. Pregnant women or those who had given birth up to three months earlier were excluded.

The following variables were considered as possible confounders: birth weight (measured by the hospital staff using pediatric scales that were calibrated weekly by the research team), maternal pregestational weight (reported by the mother in the perinatal study), gestational age (estimated from the last menstrual period), family income in minimum wages, household score index (estimated through factorial analysis and based in the ownership of household goods), and maternal schooling. Skin color was self-reported. Weight for height z-score at 4 years was estimated based on the reference population of the World Health Organization (WHO). The analyses in the 1993 cohort were also adjusted for BMI at 11 years of age.

We used Stata 12.1 for the data analysis. Means were compared using analysis of variance (ANOVA) and multiple linear regression. Outcome variables were normally distributed and variance was similar across the categories of age at menarche. Proportions were compared using the chi-squared test and prevalence ratios were estimated using Poisson regression with robust adjustments of the variance. In order to evaluate whether adiposity in late childhood confounds the association between age at menarche and body composition in adulthood, the estimates were adjusted for BMI at 11 years, in the 1993 cohort.

## Results

In 2012–13 visit, 3701 participants of the 1982 cohort were interviewed. Taking into account the 325 deaths identified among the cohort members, that represented a follow-up rate of 68.1 %. In 2011–12, 4106 participants of the 1993 cohort were interviewed, with a follow-up rate of 81.3 %, after taking into consideration the 164 deaths identified in the cohort.

Table 1 shows the characteristics of the subjects included in the analyses. Most of the subjects had a birthweight that was adequate for gestational age. The prevalence of early menarche (≤11 years of age) was 24.7 % in the 1982 cohort and 27.6 % in the 1993 cohort.

**Table 1:** Characteristics of the female population in the 1982 (N: 1637) and 1993 (N: 2033) Pelotas birth cohorts, Brazil^c^

Variables	Age at Menarche (years)					
	≤11	12–13	≥14	≤11	12–13	≥14
	1982			1993		
	N (%) or Mean (SD)					
Gestational age (w)^a^						
≤37	35 (10.9)	96 (13.6)	39 (14.1)	71 (14.3)	158 (17.1)	71 (20.3)
38–39	125 (39.1)	272 (38.4)	97 (35.2)	177 (35.6)	307 (33.2)	109 (31.1)
≥40	160 (50.0)	340 (48.0)	140 (50.7)	249 (50.1)	459 (49.7)	170 (48.6)
Birth weight (g)^a^						
<2500	37 (9.2)	75 (8.6)	27 (7.5)	58 (10.6)	103 (10.0)	57 (14.0)
2500–2999	118 (29.2)	263 (30.1)	111 (31.0)	140 (25.4)	322 (31.1)	120 (29.4)
3000–3499	145 (35.9)	316 (36.2)	138 (38.6)	236 (42.9)	380 (36.7)	157 (38.5)
≥3500	104 (25.7)	220 (25.1)	82 (22.9)	116 (21.0)	230 (22.2)	74 (18.1)
Skin color^a^						
White	290 (71.6)	666 (76.2)	275 (76.8)	355 (65.9)	653 (64.9)	264 (65.5)
Non-white	115 (28.4)	208 (23.8)	83 (23.2)	184 (34.1)	353 (35.1)	139 (34.5)
WH z-score at 4 y^b^	0.70 (0.99)	0.47 (0.95)	0.38 (0.92)	-	-	-
BMI z-score at 11 y^b^	-	-	-	0.74 (1.08)	0.24 (1.20)	−0.26 (1.22)
Age at Menarche (y)^a^	405 (24.7)	874 (53.4)	358 (21.9)	550 (27.6)	1037 (52.0)	408 (20.5)
Age at Menarche (y)^b^	10.5 (0.8)	12.4 (0.5)	14.5 (0.8)	10.6 (0.7)	12.4 (0.5)	14.4 (0.7)
BMI (kg/m^2^)^a^						
<25	123 (30.6)	440 (50.6)	202 (56.4)	347 (63.1)	732 (70.7)	336 (82.4)
25–29.9	129 (32.1)	250 (28.7)	87 (24.3)	117 (21.3)	199 (19.2)	60 (14.7)
≥30	150 (37.3)	180 (20.7)	69 (19.3)	86 (15.6)	104 (10.1)	12 (2.9)
WC (cm)^b^	84.8 (12.9)	79.9 (11.6)	78.4 (11.3)	76.0 (10.7)	73.7 (9.6)	70.9 (7.9)
AVFLT (cm)^b^	5.29 (1.91)	4.87 (1.65)	4.74 (1.41)	-	-	-
FMI (kg/m^2^)^b^	12.5 (4.7)	10.3 (4.1)	9.7 (4.2)	9.4 (3.8)	8.4 (3.5)	7.2 (3.0)
FFMI (kg/m^2^)^b^	16.4 (2.2)	15.7 (1.7)	15.6 (1.7)	15.1 (1.5)	15.0 (1.5)	14.8 (1.3)

*Abbreviation*: *SD* standard deviation, *w* weeks, *g* grams, *WH* weight for height z-score at 4 years, *y* years, *BMI* body mass index, *WC* waist circumference, *AVFLT* abdominal visceral fat layer thickness, *FMI* fat mass index, *FFMI* fat-free mass index

^a^N (%); ^b^Mean (SD) ^c^The total number of women per variable may not add up to 1637 (1982) or 2033 (1993) due to missing data

Table 2 shows that, at 30 years of age, age at menarche was negatively associated with waist circumference, visceral fat layer thickness, BMI, fat mass index, fat-free mass index, and overweight. Similar association were observed in the 1993 cohort (Table 3).

**Table 2:** Mean and confidence interval of the body composition variables at 30 years in the 1982 Pelotas birth cohort according to categories of age at menarche

Variables at 30 years	Age at Menarche (years)						*P*-value
	≤11		12–13		≥14		
	*N*	Mean	*N*	Mean	*N*	Mean	
		(95 % CI)		(95 % CI)		(95 % CI)	
Waist circumference (cm)	405	84.8	874	79.9	358	78.4	<0.0001*
		(83.5; 86.0)		(79.1; 80.6)		(77.2; 79.6)	
Abdominal visceral fat layer thickness (cm)	395	5.29	859	4.87	354	4.74	<0.0001*
		(5.10; 5.48)		(4.76; 4.98)		(4.59; 4.89)	
Fat mass index (kg/m^2^)	385	12.5	855	10.3	349	9.7	<0.0001*
		(12.1; 13.0)		(10.1; 10.6)		(9.3; 10.2)	
Fat-free mass index (kg/m^2^)	385	16.4	855	15.1	349	15.6	<0.0001*
		(16.2; 16.6)		(15.6; 15.8)		(15.4; 15.8)	
BMI (kg/m^2^)	402	29.1	870	26.2	361	25.5	<0.0001*
		(28.5; 29.7)		(25.8; 26.6)		(24.9; 26.1)	
Overweight (%)	279	69.4	430	49.4	158	43.8	<0.001*
		(64.9; 73.9)		(46.1; 52.3)		(38.6; 48.9)	

*Abbreviation: BMI* body mass index, *CI* confidence interval

*Linear trend test

**Table 3:** Mean and confidence interval of the body composition variables at 18 years in the 1993 Pelotas birth cohort according to categories of age at menarche

Variables at 18 years	Age at Menarche (years)						*P*-value
	≤11		12–13		≥14		
	*N*	Mean	*N*	Mean	*N*	Mean	
		(95 % CI)		(95 % CI)		(95 % CI)	
Waist circumference (cm)	550	76.0	1037	73.7	408	70.9	<0.0001*
		(75.1; 76.9)		(73.2; 74.3)		(70.1; 71.7)	
Fat mass index (kg/m^2^)	530	9.40	1008	8.43	402	7.19	<0.0001*
		(9.08; 9.72)		(8.22; 8.65)		(6.89; 7.49)	
Fat-free mass index (kg/m^2^)	530	15.12	1008	15.00	402	14.75	0.0009*
		(15.00; 15.25)		(14.90; 15.09)		(14.62; 14.88)	
BMI (kg/m^2^)	550	24.8	1036	23.5	408	21.9	0.0001*
		(24.4; 25.2)		(23.2; 23.8)		(21.6; 22.3)	
Overweight (%)	203	36.9	303	29.3	72	17.7	<0.001*
		(32.9; 41.0)		(26.5; 32.0)		(13.9; 21.4)	

*Abbreviation BMI* body mass index, *CI* confidence interval

*Linear trend test

Even after adjusting for possible confounding variables, age at menarche was inversely associated with waist circumference, abdominal visceral fat layer thickness, body mass index, fat mass index, fat-free mass index, and overweight prevalence at age of 30 years (Table 4).

**Table 4:** Body composition at 30 years in the 1982 Pelotas birth cohort according to categories of age at menarche

Variables at 30 years	Age at Menarche (years)			*P*-value^c^
	≤11	12–13	≥14	
	β (95 % CI)^a^			
Waist circumference (cm)	5.27	1.76	Reference	<0.0001*
	(3.43; 7.10)	(0.18; 3.33)		
Abdominal visceral fat layer thickness (cm)	0.51	0.17	Reference	<0.001*
	(0.23; 0.79)	(−0.07; 0.41)		
Fat mass index (kg/m^2^)	2.33	0.56	Reference	<0.0001*
	(1.64; 3.02)	(−0.02; 1.15)		
Fat-free mass index (kg/m^2^)	4.11	1.09	Reference	<0.0001*
	(2.74; 5.48)	(−0.08; 2.26)		
BMI (kg/m^2^)	2.98	0.81	Reference	<0.0001*
	(2.07; 3.88)	(0.03; 1.59)		
Overweight^a,b^	1.5	1.15	Reference	<0.0001**
	(1.28; 1.75)	(0.98; 1.34)		

^a^Adjusted for: maternal pregestational weight, maternal schooling, household score index, family income, birth weight, skin color, and weight for height z-score at 4 years; ^b^Prevalence Ratio (95 % CI); ^c^Linear trend test *Multi-variable regress **Poisson

Table 5 shows that in the 1993 cohort, the associations were in a similar direction to that observed in the 1982 cohort. Nevertheless, after adjusting for BMI z-score at 11 years, the magnitude of the associations decreased and the confidence interval included the reference. On the other hand, an association was still observed for fat-free mass index, and women with early menarche had lower fat-free mass index.

**Table 5:** Regression of the body composition variables at 18 years in the 1993 Pelotas birth cohort according to categories of age at menarche

Variables at 18 years	Confounding factor adjustment^a^	Confounding factor adjustment^a^ + BMI^c^ at 11 years	Confounding factor adjustment^a^	Confounding factor adjustment^a^ + BMI^c^ at 11 years	Reference
	β (95 % CI)				
	Age at Menarche (years)				
	≤11		12–13		≥14
Waist circumference (cm)	5.1	0.13	2.74	0.34	-
	(3.4; 6.3)	(−0.85; 1.1)	(1.7; 3.8)	(−0.51; 1.2)	
Fat mass index (kg/m^2^)	2.2	0.26	1.2	0.26	-
	(1.7; 2.6)	(−0.08; 0.60)	(0.78; 1.6)	(−0.04; 0.55)	
Fat-free mass index (kg/m^2^)	0.39	−0.24	0.24	−0.07	-
	(0.20; 0.58)	(−0.41; −0.07)	(0.07; 0.41)	(−0.22; 0.08)	
BMI (kg/m^2^)	2.8	0.13	1.5	0.20	-
	(2.2; 3.4)	(−0.31; 0.56)	(0.97; 2.0)	(−0.18; 0.58)	
Overweight^a,b^	2.2	1.2	1.7	1.2	-
	(1.7; 2.7)	(0.93; 1.4)	(1.3; 2.1)	(0.97–1.4)	

^a^Adjustment for confounding factors: maternal pregestational weight, maternal schooling, household score index (18 years of age), family income at birth, birth weight, and skin color (11 years of age); ^b^Prevalence Ratio (95 % CI); ^c^BMI z-score at 11 years

## Discussion

Early menarche was positively associated with adiposity in adolescence and adulthood, even after controlling for confounders. On the other hand, these associations vanished after controlling for BMI z-score at 11 years of age.

The high follow-up rates, even after 30 years and the lack of differences in the follow-up rate according to age at menarche suggests that selection bias is unlikely. Moreover, the short recall time of the information on age at menarche decreased the likelihood of non-differential misclassification. Because information on BMI in early adolescence was available in the 1993 cohort, it was possible to test whether the association between age at menarche and body composition in adulthood was explained by the association between age at menarche and body composition in puberty.

A higher BMI [10, 21–25], waist circumference [25, 26], total fat percentage [21, 24, 27], and visceral and subcutaneous fat [25] have been reported among women who had early menarche, even after adjusting for sociodemographic variables – age [23–27], race [23, 24], education [24, 26], parity [26]; behavioral factors – daily energy expenditure, total daily fruit and vegetable consumption [23], smoking [25], alcohol intake [25], physical activity [24, 25, 27]; and hormone replacement therapy [25], regardless of the study design.

The fetal programming hypothesis suggests that an intrauterine stimuli or aggression can induce metabolic and physiologic changes in the offspring that would increase the risk of chronic diseases in adulthood [28]. In the present study, adjustment for maternal pregestational weight and birth weight did not change the estimates on the association between early age at menarche and body composition. Our findings are in agreement with other studies that suggested that prenatal factors had little impact on sexual maturation [29] in relation to those related to childhood and adolescence body composition [30].

Concerning the mechanisms underlying the association between early menarche and body composition in adulthood. Initially, it was suggested that early menarche would program changes in body composition through hormonal action [5–7], and adolescent adiposity would remain until adulthood [31]. For example, serum levels of sexual steroids increase among women who matured early [32, 33], beyond the amount secreted by the ovaries. This increased hormone secretion occurs due to endocrinal activity of the fatty tissue, which is excessive in these women [34, 35]. Besides, the increase in the level of these hormones leads to greater body fat deposition, which remains until adulthood [32].

The increase in adiposity in the puberty would be another mechanism. In pre-menarche, the girls must acquire some adiposity [36, 37] and the greater amount of fat deposited in subcutaneous tissues in this period would be related to early menarche [38, 39]. And, adiposity in late childhood is positively related to body fat in adulthood [11, 17, 40].

It is difficult to disentangle the effect of adiposity in the late childhood from that from sexual maturity. The adjustment for BMI at 11 years, which highlight the adiposity rebound in late childhood, is a strategy to evaluate whether the association between early menarche and body composition is independent of body composition in late childhood. If age at menarche was associated with body composition in adulthood, independently of adiposity in late childhood, the estimates after controlling for adiposity in late childhood should remain unchanged. However, in the present analysis, the differences in the measures of adiposity between those women who presented a menarche in the age category 12 – 13 years and those whose menarche was at ≥14 years of age vanished after controlling for body mass index at 11 years.

## Conclusions

The findings from the analysis carried out in the 1993 cohort suggest that the association between age at menarche and body composition in adulthood is strongly related to body composition in late childhood. Therefore, age at menarche would be a proxy of pre-pubertal body composition.

## Abbreviations

ANOVA: Analysis of variance
BMI: Body mass index
DXA: Dual-energy x-ray absorptiometry
NHANES: National health and nutrition examination survey
OR: Odds ratio
SD: Standard deviation
WHO: World Health Organization
95 % CI: 95 % confidence interval
